# Educational gaps and factors associated with artificial intelligence adoption among Egyptian periodontists: a multicenter cross-sectional study

**DOI:** 10.1038/s41598-026-65434-3

**Published:** 2026-08-10

**Authors:** Hala A. Abuel Ela, Radwa Moustapha, Amira Badran, Asmaa Abou-Bakr, Hadeel Gamal Almalahy

**Affiliations:** 1 Oral Medicine and Periodontology, Ain Shams University in Egypt, Cairo, Egypt; 2 Department of Oral Medicine, Periodontology, and Oral Diagnosis, Faculty of Dentistry, Misr International University, Cairo, Egypt; 3 Pediatric Dentistry and Dental Public Health, Ain Shams University in Egypt, Cairo, Egypt; 4 Pediatric Dentistry and Dental Public Health, Faculty of Dentistry, Galala University, Suez, Egypt; 5 Oral Medicine and Periodontology, Faculty of Dentistry, Galala University, Suez, Egypt

**Keywords:** Artificial intelligence, Perception, Periodontists, Knowledge, AI applications, Periodontology, Health care, Medical research

## Abstract

In periodontology, Artificial Intelligence (AI) applications, ranging from radiographic evaluation to outcome prediction, are emerging. However, their adoption is impacted by practitioners’ awareness, attitudes, and perceived barriers. Evidence regarding AI adoption among Egyptian periodontists remains limited. Therefore, this study aimed to assess knowledge, perceptions, usage, and concerns regarding AI among Egyptian periodontists, and to identify demographic and professional factors associated with AI adoption. A multicenter cross-sectional online survey using a 33-item questionnaire was uploaded via Google Forms and distributed to eligible Egyptian periodontists. To gather information about participants’ knowledge, opinions, and concerns about artificial intelligence in periodontology, the survey used closed-ended questions with a 3-point Likert-type scale. A total of 275 Egyptian periodontists took part. Although familiarity with AI was high (98.2%), only 31.3% reported understanding its working principles. Attitudes toward AI were generally positive, with 89.8% considering it a new era, and 80.7% expecting it to significantly improve periodontology. Despite substantial interest, practical familiarity with AI-based dental software remained limited (10.9%), with research being the most common application area (58.2%), followed by implant planning (13.1%) and diagnosis (12.7%). The main concerns centered on over-reliance on AI affecting critical thinking skills (68.4%), the reliability of AI-assisted periodontal diagnosis (65.8%), security risks (59.6%), and patient privacy issues (53.1%). Ordinal logistic regression analyses identified several factors significantly associated with AI-related outcomes, with AI working principle knowledge associated with age and professional experience. Male gender was significantly associated with the perception that AI could replace periodontists. AI-related educational engagement was associated with age, professional experience, and institutional affiliation. AI-related concerns were associated with gender and educational level. The findings suggest a gap between high awareness and limited adoption of AI among Egyptian periodontists despite generally positive attitudes. Key demographic and professional variables such as age, experience, gender, and institutional affiliation emerged as significant associated factors in the regression models for knowledge, perception, usage, and concerns. The study results highlight potential educational gaps and support the integration of AI education in postgraduate programs.

**Supplementary Information:**

The online version contains supplementary material available at 10.1038/s41598-026-65434-3.

## Introduction

The potential applications of AI in dentistry are rapidly advancing^1–3^, with AI-based algorithms anticipated to increase diagnostic accuracy by addressing the limitations of existing radiographic methods^4–6^.

AI is also attracting interest in the field of periodontology^7,8^. It was proposed that using AI-driven tools to evaluate intraoral images, clinical charts, and radiographs for the identification of gingival inflammation, staging and grading of periodontitis, and detection of bone loss would facilitate the diagnosis, treatment, and prognostic prediction of periodontal disorders^9,10^.

These AI-driven techniques support earlier and more objective disease detection by addressing major drawbacks of traditional diagnostic techniques, such as subjectivity, time constraints, and inter-examiner variability^11^. While AI has various potential benefits, it has received mixed responses regarding the advantages and risks associated with advanced AI technologies^1,12,13^. Additionally, security concerns and the potential of cyberattacks with the spread of misinformation utilizing AI should also be considered^14–16^. To ensure the safe and successful integration of AI into periodontal care, it is crucial to understand practitioners’ acceptance of this technology.

Earlier international studies and reviews have examined the integration of AI in periodontics, with a particular emphasis on its potential for diagnosis and treatment planning^17–20^. However, only one previous study has evaluated periodontists’ understanding, attitudes, and concerns about the use of AI globally^21^. There is a lack of evidence specifically focusing on Egyptian periodontists. This is a crucial gap since the adoption of AI may be significantly impacted by regional variations in clinical training, access to digital infrastructure, and cultural views of technology. Furthermore, as specialized clinicians, periodontists are uniquely positioned at the forefront of adopting diagnostic innovations^22^, whereas the majority of prior studies focused on dental students or general practitioners.

From an educational perspective, the inclusion of artificial intelligence in dental curricula and postgraduate training programs is necessary to train future professionals to work with AI assistance. The applications of AI in periodontal education consist of AI-enabled diagnosis, radiographic and clinical periodontal data interpretation, treatment planning, simulated learning, and clinical decision-support systems that can be used to improve postgraduate education.

According to current research, despite the positive attitude of dental care providers towards AI technology, limited knowledge of AI principles, insufficient practical experience with AI-based tools, and uncertainty regarding ethical and clinical uses of AI technology remain major barriers to successful implementation.

Consequently, the assessment of the readiness of specialists and their educational needs regarding AI technology is crucial before its integration into periodontal practice. Nevertheless, there is a lack of data on specialists’ preparedness and related educational needs, especially in developing countries. Although previous articles have been published about the use of artificial intelligence in dentistry, most of them have been based primarily on dental students and general dentists; however, evidence regarding periodontists remains limited. This is an important gap since periodontists directly implement the use of advanced technologies in periodontal assessment, treatment planning, and prediction of periodontal prognosis. Although there is no direct assessment of dental curricula through clinicians’ opinions, an assessment of periodontists’ knowledge and experience with AI can provide valuable insight into educational needs and readiness in this regard.

Therefore, this study aimed to assess the knowledge, perception, usage, and concerns related to AI among Egyptian periodontists, and to detect demographic and professional factors associated with these outcomes. In addition to being nationally relevant, filling this gap adds to the global debate about the ethical and successful integration of AI into dental education and specialty practice. Unlike previous mainly descriptive studies, this study employs multivariate regression analysis to identify factors associated with AI adoption among specialists. We hypothesized that variables such as demographic and professional factors are independently associated with AI knowledge, perception, usage, and concerns among Egyptian periodontists.

## Methods

### Study design

This was an exploratory, multicenter cross-sectional study using a self-administered online questionnaire conducted among a group of periodontists from multiple academic dental institutions, including public and private universities across Egypt. The participating institutions included Ain Shams University (ASU), Cairo University (CU), Al-Azhar University (AZHAR), the British University in Egypt (BUE), Misr International University (MIU), Badr University in Cairo (BUC), Future University in Egypt (FUE), and Galala University (GU).

The study was conducted based on the Checklist for Reporting Results of Internet E-Surveys (CHERRIES) to ensure proper reporting of the online survey methodology as presented in Supplementary File 1.

This study was multicenter in nature as participants were recruited from several academic institutions instead of a single one, while the data were collected centrally using an online survey.

The survey structure was adapted from Chawla et al.^21^, comprising 33 closed-ended questions in English on a 3-point Likert scale (Yes/Maybe/No) to minimize neutral bias^21^. This study is reported in accordance with the STROBE (Strengthening the Reporting of Observational Studies in Epidemiology) guidelines for cross-sectional studies.

*Study timeline*: The questionnaire was distributed to the eligible participants. This study took place during the academic year (2023–2024).

*Ethical aspects*: The study methodology was reviewed and approved by the research ethics committee at the Faculty of Dentistry, Ain Shams University, Egypt (approval number FDASU-RECIM012404). All methods were performed in accordance with the relevant guidelines and regulations and the Declaration of Helsinki.

Volunteers who satisfied the following study inclusion and exclusion criteria were informed about the purpose of the study, and informed consent was requested at the start of participation to answer the online survey questionnaire while maintaining their anonymity and confidentiality according to the survey’s settings^23^.

*Sample size estimation*: The sample size required was determined through a two-stage process. First, to achieve the primary descriptive results, 207 were determined using a standard formula for proportion estimation (95% CI, with 5% margin of error). To account for the anticipated non-response rate of 30%, we sought to recruit 275 participants. Then, the achieved final sample size of 275 was assessed for adequacy in advanced multivariate analysis. The sample size post-hoc power analysis done in G*Power, version 3.1, for logistic regression considering five predictors showed that this sample size gave > 80% power detecting a small-to-medium effect size, represented as an odds ratio = 1.5 at an α = 0.05 level of significance^21,24^.

Participants’ eligibility criteria:

The survey was distributed widely to eligible Egyptian periodontists, yielding 275 complete responses:

*Inclusion criteria*:

1. Post-graduate periodontists.
2. Egyptian periodontists.

*Exclusion criteria*:

1. Undergraduate dental student.
2. Periodontists who declined participation or did not provide informed consent.

To provide a representative sample of the national dental education framework, the study sample was limited to Egyptian periodontists. This method reduces the disparity that arises between periodontists from other nations or school systems due to disparate clinical training, curriculum, and access to AI technologies.

*Sampling Strategy*: A non-probability convenience sampling approach was employed for participant recruitment due to the absence of a comprehensive national registry of practicing periodontists and the exploratory nature of the study. Potential participants from the Egyptian periodontist population were recruited by invitation through emails and digital communication means.

The sampling process was performed mainly using the official mailing list of the Egyptian Society of Periodontology and postgraduate periodontology programs in Egyptian universities, in order to ensure diversity in institutions and locations. Participants had to respond voluntarily and anonymously to the survey, which stayed open for participation until the sample size was exceeded by the number of completed surveys.

Even though such a recruitment strategy ensured an efficient and effective recruitment process, it is likely that it will result in bias, and the findings will have low generalizability.

Despite the use of convenience sampling, efforts were made to ensure diversity across institutions, improving representativeness.

*Outcomes measure*:

The study aimed to assess five primary outcomes:

1. Periodontists’ knowledge about AI.
2. Periodontists’ perception of AI and its role in dental practice.
3. The extent and nature of AI usage among periodontists.
4. Concerns and perceived barriers related to AI adoption in dental education and clinical practice.
5. The relationship between demographic variables and AI-related knowledge, perceptions, usage, and concerns.

### Data sources and measurement

Data were collected using a pre-prepared, self-administered English questionnaire adapted from the validated questionnaire developed by Chawla et al.^21^. The questionnaire was further refined using evidence from previous validated studies^25,26^ to ensure its relevance to the study objectives. A pilot test was conducted with 20 Egyptian periodontists (not included in the main sample) to assess the clarity, feasibility, and comprehension. Most respondents reported that the instrument was clear (90%), easy to comprehend (95%), and well-timed (95%). There were no serious changes necessary.

Scoring was done by means of a three-point Likert scale, where Yes = 3, Maybe = 2, No = 1.

*Reliability and validity*:

The internal consistency reliability of the domains of the questionnaire was established using Cronbach’s alpha coefficients. The results revealed that the questionnaire had adequate reliability regarding perceptions (α = 0.72) and concerns (α = 0.77), while moderate reliability was reported for knowledge (α = 0.64).

The electronic survey, constructed using Google Forms, was sent to periodontists fulfilling the eligibility criteria.

Participation was entirely voluntary; no personal information was collected to ensure the confidentiality and anonymity of the participants. Before proceeding with the questionnaire, each participant gave informed consent by answering a (Yes/No) question. Participants who gave consent were allowed to proceed with completing the questionnaire. To ensure data privacy and confidentiality, the online questionnaire did not collect any personally identifiable information, and all responses were recorded anonymously. Access to the collected data was restricted to the research team, and the data were used only for research purposes. Cookies were not used to identify participants or track survey responses. IP addresses were not collected, stored, or used to identify participants.

This questionnaire comprised five sections with 33 questions, as presented in the Supplementary file.

The survey was created as an obligatory response questionnaire to guarantee data completeness. Participants were required to complete all questions before submission, guaranteeing that no answers were omitted or lacking. Before final submission of the questionnaire, participants were able to review their responses and modify their answers if needed.

*Questionnaire Tool*: 

Data were collected through a structured questionnaire consisting of multiple Sect^21^:

The questionnaire was delivered to periodontists as it was voluntary and anonymous^27^. The questionnaire consisted of five parts that aimed to provide information on perceptions of artificial intelligence in dental practice, knowledge, usage, and concerns. The average time to complete the questionnaire is 10–12 min.

*Demographic Data*: The first section, known as **Part A**, focused on five open-ended questions to assess the following dimensions of the respondents: demographic characterization (gender and age), and professional characterization (academic affiliation, years of experience, and university of graduation).

*Knowledge of AI*^21^: **Part B** contained a list of structured questions on periodontists’ basic knowledge of AI on a Likert three-point scale (yes/no/maybe)^23^.

*AI perception*:** Part C** explored how periodontists perceive the possibilities of AI implementation, using a 3-point Likert scale (yes/no/maybe). Questions centered on thoughts about whether AI will revolutionize dentistry, its potential for benefits around productivity, accuracy, and patient care, and concerns regarding whether AI could replace human dentists one day. It also assessed their attitudes regarding AI as an ephemeral trend in healthcare and whether they were interested in learning about AI concepts.

*Usage of AI*:** Part D** consisted of questions focusing on the usage of AI applications. They were asked if they had ever used AI applications, how long they had been using them, what kind of AI software they had used, and whether they had taken part in any workshops, webinars, or seminars pertaining to AI education. The section also identified gaps in AI education and professional exposure.

*Concerns About AI*:** Part E** consisted of questions assessing concerns about AI applications in periodontology using a Likert three-point scale (yes /no / maybe). The evaluation included concerns about the accuracy and reliability of AI, its impact on clinical judgment, ethical implications, over-reliance on technology, potential loss of research originality, and the lack of clinical validation for AI applications^28^. It also looked at perceived obstacles to AI adoption, namely, practitioners’ and patients’ lack of understanding.

The scoring system **(Yes = 3**,** Maybe = 2**,** and No = 1)** was adopted to reflect increasing levels of agreement and certainty. The inclusion of “Maybe” intentionally positions this option as the middle response to avoid forced dichotomization and capture uncertainty in emerging concepts such as AI. A 4-point Likert scale was utilized in the case of measuring the level of agreement on the statement concerning how AI could minimize iatrogenic errors because this particular question was aimed at measuring the level of agreement rather than concern.

Categorization thresholds (< 50%, 50–75%, > 75%) were identified from previously published KAP-based survey studies that defined low, moderate, and high levels of knowledge, perception, usage, and concerns; however, these cut-off values were used to facilitate interpretation of the findings and were not considered universal standardized criteria^29^.

*Bias Control*: Although the survey was widely distributed, the use of convenience sampling may introduce selection bias and limit representativeness. To minimize information bias, the objective of the study and instructions were clearly presented to all participants^30^. To reduce the impact of social desirability bias, participation was entirely voluntary, and anonymity was ensured. All participants received uniform and clear explanations of the study’s purpose and instructions in order to reduce information bias. Standardized wording was used to administer the survey in English, and a pilot test ensured the questions were clear. Additionally, Google Forms was set up with mandatory responses, which removed missing information and produced comprehensive datasets.

### Statistical analysis of the data

Data were entered into the computer and analyzed using the IBM SPSS software package version 27.0. (Armonk, NY: IBM Corp, released 2020). Categorical data were represented as numbers and percentages. The Chi-square test was applied to assess associations between demographic and professional variables (gender, age, educational level, and institutional affiliation) and categorized AI-related outcomes (knowledge, perception, usage, and concerns). Alternatively, Fisher’s Exact test or Monte Carlo correction was applied when more than 20% of the cells had expected counts less than 5.

Ordinal logistic regression was performed to identify demographic and professional factors independently associated with AI knowledge, perception, usage, and concerns. Significance of the obtained results was judged at the 5% level. The proportional odds assumption was tested using the test of parallel lines (*p* > 0.05). Model fit was assessed using the likelihood ratio χ² and Nagelkerke pseudo-R². Variables included in the regression models were selected based on theoretical relevance and prior literature.

## Results

### Demographic data of the participants

The sample consisted of 275 participants, with a slightly higher proportion of females (54.5%) than males (45.5%). The mean age was 30.69 ± 4.83 years, and the majority of participants (61.1%) belonged to the 30–<35-year age group.

The highest educational attainment for the majority was a Master’s degree (80.7%), while 19.3% had a PhD. Regarding professional experience, 67.6% had ≤ 5 years of experience, and 32.4% had > 5 years. Participants were recruited from both public and private universities, representing different academic institutions across Egypt. The sample was roughly evenly divided between participants affiliated with public universities (50.9%) and private universities (49.1%) (Table 1).

**Table 1:** Distribution of the studied participants according to demographic characteristics, educational level, professional experience, and institutional affiliation (*n* = 275).

Demographic data	No. (%)
Gender	
Male	125 (45.5%)
Female	150 (54.5%)
Age (years)	
< 30	107 (38.9%)
30 – <35	168 (61.1%)
** Min. – Max.**	22.0–66.0
** Mean ± SD.**	30.69 ± 4.83
** Median (IQR)**	30.0 (29.0–32.0)
Level of education	
MSc	222 (80.7%)
PhD	53 (19.3%)
Faculty	
** Public universities**	**140 (50.9%)**
ASU	94 (34.2%)
CU	34 (12.4%)
AZHAR	12 (4.4%)
** Private universities**	**135 (49.1%)**
BUE	47 (17.1%)
MIU	49 (17.8%)
BUC	6 (2.2%)
FUE	13 (4.7%)
GU	20 (7.3%)

IQR: Interquartile range SD: Standard deviation.

### Knowledge of artificial intelligence

Participants demonstrated varying levels of knowledge, with 47.6% showing a high level of knowledge, 51.3% showing an average level, and 1.1% showing a low level.

Concerning more unique applications of AI in periodontology, nearly half of the participants believed in the potential of AI applications in the diagnosis of the staging and grading of periodontitis (51.3%), detection of periodontal bone loss (56.4%), assessment of the prognosis of questionable teeth (50.5%), and prediction of the clinical attachment levels (48.0%) (Table 2).

**Table 2:** Distribution of the studied cases according to knowledge of (AI) (*n* = 275).

Q	Knowledge of AI	Yes	No	Maybe
1	Are you aware of the term ‘artificial intelligence (AI)?	270 (98.2%)	2 (0.7%)	3 (1.1%)
2	Are you aware of the working principle of AI?	86 (31.3%)	90 (32.7%)	99 (36.0%)
3	Can AI diagnose cases of different staging and grading of periodontitis?	141 (51.3%)	8 (2.9%)	126 (45.8%)
4	Can AI be used in the clinical diagnosis of periodontal bone loss?	155 (56.4%)	12 (4.4%)	108 (39.3%)
5	Can AI assess the prognosis of questionable teeth?	139 (50.5%)	14 (5.1%)	122 (44.4%)
6	Can AI predict clinical attachment levels?	132 (48.0%)	16 (5.8%)	127 (46.2%)

### Perception of artificial intelligence

The attitudes towards AI tended to be highly positive. Most participants viewed AI as the beginning of a new era in dentistry (89.8%), while (80.7%) believed its emergence would improve periodontology significantly.

Most participants favored its incorporation into the curriculum in postgraduate courses (82.9%) and in its use in the clinic for radiographic image analysis (82.5%).

However, 69.8% did not believe AI surpasses human diagnostic ability, and 78.2% rejected the idea that AI could replace a periodontist in the future (Table 3).

**Table 3:** Distribution of the studied cases according to perception of AI (*n* = 275).

Q	Perception of artificial intelligence (AI)	Yes	No	Maybe
1	Do you think AI is a new era in dentistry?	247 (89.8%)	10 (3.6%)	18 (6.5%)
2	Do you think AI is just a new trend?	20 (7.3%)	201 (73.1%)	54 (19.6%)
3	Do you think periodontology is adequately equipped for applications of AI?	132 (48.0%)	34 (12.4%)	109 (39.6%)
4	Do you think that the “introduction of Artificial Intelligence in periodontology will lead to a huge improvement in this field ”?	222 (80.7%)	6 (2.2%)	47 (17.1%)
5	Do you think AI has a better diagnostic ability than periodontists?	17 (6.2%)	192 (69.8%)	66 (24.0%)
6	Do you think AI could replace periodontists in the future?	14 (5.1%)	215 (78.2%)	46 (16.7%)
7	Do you think AI should be an integral part of postgraduate training?	228 (82.9%)	9 (3.3%)	38 (13.8%)
8	Do you think AI-based software should be used in clinical practice for ease in the radiographic analysis of periodontal diseases?	227 (82.5%)	4 (1.5%)	44 (16.0%)
9	Do you think AI improves the efficacy of periodontal therapy?	166 (60.4%)	8 (2.9%)	101 (36.7%)
10	Do you think AI could improve the predictability of periodontal disease prognosis?	162 (58.9%)	9 (3.3%)	104 (37.8%)
11	Do you think AI can improve patient satisfaction concerning periodontal therapy?	162 (58.9%)	12 (4.4%)	101 (36.7%)
12	Do you think that AI software can improve periodontal surgery outcomes?	142 (51.6%)	16 (5.8%)	117 (42.5%)

### Usage of artificial intelligence

Actual use of AI was limited, despite the high awareness. The level of familiarity with AI-based dental software was (10.9%), although interest among non-users was (74.9%). Similarly, while 40.4% of the study group had taken part in a webinar/session on AI, a large group (52.0%) of those who didn’t were interested.

The major fields of application of AI were research (58.2% of participants), followed by implant planning (13.1%), and diagnosis (12.7%). The usage of AI occurred monthly (45.5%), though (29.1%) had never used it (Table 4).

**Table 4:** Distribution of the studied cases according to usage of artificial intelligence (AI) (*n* = 275).

Q	Usage of artificial intelligence (AI)	No. (%)
1	Have you ever attended any webinar/lecture/course on Artificial Intelligence in healthcare?	
	If No : Interested	143 (52.0%)
	No	21 (7.6%)
	Yes	111 (40.4%)
2	Do you use or are you familiar with the usage of any of the current applications of AI-based dental software?	
	If No : Interested	206 (74.9%)
	No	39 (14.2%)
	Yes	30 (10.9%)
3	Which resource have you used the most to learn about Artificial Intelligence and its applications?	
	Formal training (e.g., courses) in Artificial Intelligence	4 (1.5%)
	Friends & Colleagues	90 (32.7%)
	Journal, articles & books	15 (5.5%)
	Medical school lectures	53 (19.3%)
	Nothing	15 (5.5%)
	Others	4 (1.5%)
	Webinars	15 (5.5%)
	Websites	79 (28.7%)
4	Have you used AI for research purposes in periodontics?	
	If No : Interested	53 (19.3%)
	No	53 (19.3%)
	Yes	169 (61.5%)
5	Have you used AI in diagnosing periodontal problems?	
	If No : Interested	149 (54.2%)
	No	56 (20.4%)
	Yes	70 (25.5%)
6	How often do you use artificial intelligence in your daily work?	
	Monthly	125 (45.5%)
	Never	80 (29.1%)
	Weekly	70 (25.5%)
7	What are the areas of application you use AI for?	
	Diagnosis	35 (12.7%)
	Implant	36 (13.1%)
	None	37 (13.5%)
	Research	160 (58.2%)
	Treatment	7 (2.5%)
8	In comparison to your colleagues in your profession, how would you rate your knowledge of the topic of AI and its application possibilities in your profession?	
	Above Average	5 (1.8%)
	Average	230 (83.6%)
	Below Average	40 (14.5%)
9	How long will it take, in your opinion, until AI has a noticeable impact on your profession?	
	Never	4 (1.5%)
	< 1 year	46 (16.7%)
	1–5 years	90 (32.7%)
	5–10 years	65 (23.6%)
	> 10 years	70 (25.5%)
10	Can you imagine implementing the following workflow in your Clinical life: Radiographs of a patient are diagnosed by an AI?	
	Yes	215 (78.2%)
	No	11 (4.0%)
	Maybe	49 (17.8%)

### Concerns about AI usage

Most participants were concerned about the accuracy of AI-assisted diagnoses of periodontal diseases (65.8%) and the potential impact on the development of critical thinking among professionals (68.4%). Concerns also included potential AI-related security risks (59.6%) and the possible violation of patient privacy (53.1%) (Table 5).

**Table 5:** Distribution of the studied cases according to concerns about AI usage (*n* = 275).

Q	Concerns about AI usage	No. (%)
1	Are you concerned about the reliability of the periodontal diagnosis provided by AI?	
	Yes	181 (65.8%)
	No	11 (4.0%)
	Maybe	83 (30.2%)
2	Are you afraid that relying too much on AI and not developing critical thinking skills of the periodontist?	
	Yes	188 (68.4%)
	No	22 (8.0%)
	Maybe	65 (23.6%)
3	Are you concerned about the potential security risk of using AI in periodontology?	
	Yes	164 (59.6%)
	No	30 (10.9%)
	Maybe	81 (29.5%)
4	Are you afraid that the use of AI would be a violation of patient privacy?	
	Yes	146 (53.1%)
	No	55 (20.0%)
	Maybe	74 (26.9%)
5	To what extent do you agree with the following statement: “The introduction of AI will reduce iatrogenic errors in my profession.”	
	Strongly Disagree	2 (0.7%)
	Disagree	27 (9.8%)
	Agree	156 (56.7%)
	Strongly agree	90 (32.7%)

### Feedback from questionnaire

The questionnaires were well-received, with a very high percentage feeling that the time was appropriate (97.8%), the questions were clear enough (97.1%), and the questions contained in the form were easy to understand (98.9%).

### Association between gender and AI-related domains

A statistically significant association was established only between gender and the usage of AI (*p* < 0.001). Female participants demonstrated higher proportions of high AI usage, whereas male participants were more likely to report moderate levels of usage. Non-significant results were observed between gender and levels of knowledge (*p* = 0.499), perception (*p* = 0.277), and concerns (*p* = 0.828), indicating comparable levels across males and females (Table 6).

**Table 6:** Association between Gender and Level of Knowledge-based questions, Perception, Usage, and Concerns about AI usage (n = 275).

	Gender		χ^2^	*p*
	Male (*n* = 125)	Female (*n* = 150)		
**Knowledge-based questions**				
Low knowledge	2 (1.6%)	1 (0.7%)	1.472	^MC^p= 0.499
Average knowledge	60 (48.0%)	81 (54.0%)		
High knowledge	63 (50.4%)	68 (45.3%)		
**Perception of AI**				
Negative perception	12 (9.6%)	10 (6.7%)	2.569	0.277
Neutral perception	47 (37.6%)	70 (46.7%)		
Positive perception	66 (52.8%)	70 (46.7%)		
**Usage of AI**				
Low usage of AI	12 (9.6%)	33 (22.0%)	17.030^*^	< 0.001^*^
Moderate usage of AI	81 (64.8%)	61 (40.7%)		
High usage of AI	32 (25.6%)	56 (37.3%)		
**Concerns about AI usage**				
Low risk	3 (2.4%)	3 (2.0%)	0.424	^MC^p= 0.828
Moderate risk	52 (41.6%)	58 (38.7%)		
High risk	70 (56.0%)	89 (59.3%)		

χ^2^: Chi square test MC: Monte Carlo.

p: p value for Association between AI-related outcomes and demographic variables.

*Statistically significant at *p* ≤ 0.05.

### Association between age and AI-related domains

Age group showed significant associations with knowledge of AI (*p* = 0.001) and usage of AI (*p* < 0.001), as well as concerns regarding AI usage (*p* = 0.023). No significant association was observed between age group and perception of AI (*p* = 0.170) (Table 7).

**Table 7:** Association between Age group with Level of Knowledge-based questions, Perception, Usage, and Concerns about AI usage (n = 275).

	Age group (Years)		χ^2^	*p*
	< 30 (*n* = 107)	≥ 30 (*n* = 168)		
Knowledge-based questions				
Low knowledge	2 (1.9%)	1 (0.6%)	12.542^*^	^MC^p= 0.001^*^
Average knowledge	68 (63.6%)	73 (43.5%)		
High knowledge	37 (34.6%)	94 (56.0%)		
**Perception of AI**				
Negative perception	8 (7.5%)	14 (8.3%)	3.550	0.170
Neutral perception	53 (49.5%)	64 (38.1%)		
Positive perception	46 (43.0%)	90 (53.6%)		
**Usage of AI**				
Low usage of AI	31 (29.0%)	14 (8.3%)	31.641^*^	< 0.001^*^
Moderate usage of AI	59 (55.1%)	83 (49.4%)		
High usage of AI	17 (15.9%)	71 (42.3%)		
**Concerns about AI usage**				
Low risk	5 (4.7%)	1 (0.6%)	7.317^*^	^MC^p= 0.023^*^
Moderate risk	48 (44.9%)	62 (36.9%)		
High risk	54 (50.5%)	105 (62.5%)		

χ^2^: Chi square test MC: Monte Carlo.

p: p value for Association between AI-related outcomes and demographic variables.

*Statistically significant at *p* ≤ 0.05.

### Association between the level of education and AI-related domains

The results show that PhD holders possess a significantly higher level of AI knowledge (*p* = 0.001) and a higher positive perception of AI (*p* = 0.009) compared to the MSc holders. No significant associations were found between the level of education and AI usage (*p* = 0.677) or concerns (*p* = 0.061) (Table 8). This may reflect greater academic exposure and research engagement among PhD holders.

**Table 8:** Association between Level of education with Level of Knowledge-based questions,Perception, Usage, and Concerns about AI usage (n = 275).

	Level of education		χ^2^	*p*
	MSc (*n* = 222)	PhD (*n* = 53)		
**Knowledge-based questions**				
Low knowledge	2 (0.9%)	1 (1.9%)	12.334^*^	^MC^p= 0.001^*^
Average knowledge	125 (56.3%)	16 (30.2%)		
High knowledge	95 (42.8%)	36 (67.9%)		
**Perception of AI**				
Negative perception	15 (6.8%)	7 (13.2%)	9.413^*^	0.009^*^
Neutral perception	104 (46.8%)	13 (24.5%)		
Positive perception	103 (46.4%)	33 (62.3%)		
**Usage of AI**				
Low usage of AI	38 (17.1%)	7 (13.2%)	0.781	0.677
Moderate usage of AI	112 (50.5%)	30 (56.6%)		
High usage of AI	72 (32.4%)	16 (30.2%)		
**Concerns about AI usage**				
Low risk	6 (2.7%)	0 (0.0%)	5.205	^MC^p= 0.061
Moderate risk	95 (42.8%)	15 (28.3%)		
High risk	121 (54.5%)	38 (71.7%)		

χ^2^: Chi-square test MC: Monte Carlo.

p: p-value for Association between AI-related outcomes and demographic variables.

*Statistically significant at *p* ≤ 0.05.

### Association between faculty type and AI-related domains

A statistically significant association was established with the use of AI (p < 0.001). Participants from public universities showed higher rates of ‘High usage’ (39.3% vs. 24.4%), while those from private universities showed higher ‘Moderate usage’ (63.7% vs. 40.0%). There are no significant associations between faculty type and the levels of knowledge (*p* = 0.183), perception (*p* = 0.406), or concerns (*p* = 0.291) (Table 9).

**Table 9:** Association between Faculty with Level of Knowledge-based questions, Perception, Usage, and Concerns about AI usage (n = 275).

	Faculty		χ^2^	*p*
	Public universities (*n* = 140)	Private universities (*n* = 135)		
**Knowledge-based questions**				
Low knowledge	1 (0.7%)	2 (1.5%)	3.285	^MC^p= 0.183
Average knowledge	79 (56.4%)	62 (45.9%)		
High knowledge	60 (42.9%)	71 (52.6%)		
**Perception of AI**				
Negative perception	10 (7.1%)	12 (8.9%)	1.801	0.406
Neutral perception	65 (46.4%)	52 (38.5%)		
Positive perception	65 (46.4%)	71 (52.6%)		
**Usage of AI**				
Low usage of AI	29 (20.7%)	16 (11.9%)	15.508^*^	< 0.001^*^
Moderate usage of AI	56 (40.0%)	86 (63.7%)		
High usage of AI	55 (39.3%)	33 (24.4%)		
**Concerns about AI usage**				
Low risk	2 (1.4%)	4 (3.0%)	2.651	^MC^p= 0.291
Moderate risk	62 (44.3%)	48 (35.6%)		
High risk	76 (54.3%)	83 (61.5%)		

χ^2^: Chi-square test MC: Monte Carlo.

p: p-value for Association between AI-related outcomes and demographic variables.

*Statistically significant at *p* ≤ 0.05.

### The regression analysis

Outcome variables were categorized into ordinal levels (low, moderate, high) based on predefined scoring thresholds.

### Multivariate ordinal logistic regression analysis

Age category and professional experience showed significant associations with knowledge of AI working principles (Supplementary Table 1). According to the coding direction of the ordinal regression model, both age category and professional experience were significantly associated with AI knowledge levels (OR = 32.33; *p* = 0.002 and OR = 11.61; *p* = 0.009, respectively). Nevertheless, the wide confidence intervals indicate possible model instability; thus, these findings should be interpreted cautiously.

Male gender was significantly associated with the perception that AI would replace periodontists (OR = 7.87, *p* = 0.026), Supplementary Table 2.

As for the variables associated with the use of AI, age, professional experience, and institutional affiliation appeared as important associated factors for AI-related engagement in education, while research-related AI use was mainly associated with age and professional experience, Supplementary Table 3.

As for the concerns, male respondents expressed fewer concerns regarding the accuracy of the diagnosis (OR = 0.197, *p* = 0.030), while PhD holders showed greater concern regarding patient privacy compared with MSc holders, as MSc participants had lower odds of reporting this concern (OR = 0.209, *p* = 0.016), Supplementary Table 4.

## Discussion

This study presents one of the first comprehensive assessments of the knowledge, perception, and usage of AI among periodontists in Egypt, with particular attention to demographic and professional factors associated with these outcomes.

Although the transformative potential of AI in periodontology is widely recognized^7–9^, the pathway toward its clinical integration remains poorly understood, especially in underrepresented parts of the world.

Our findings indicate a discrepancy between high awareness and limited practical adoption: an overwhelming majority (98.2%) of periodontists are aware of AI, yet only a small fraction of them (10.9%) actively use it in their practice. This significant gap between high awareness and low usage underlines a critical transition period in which potential has not yet been translated into common practice, highlighting a key area for intervention.

### Knowledge and perception in context

Regarding foundational knowledge, only 31.3% of our participants reported knowing the working principles of AI. This number is lower compared to the percentages reported among dental professionals in Saudi Arabia, at 50.1%^31^, and India, at 73.70%^32^, but comparable to that from Moroccan students, at 48.40%^35^. The variability in the integration of AI topics into dental curricula may account for this discrepancy^23^.

Despite this lack of foundational knowledge, there was considerable optimism regarding the role of AI in the future of periodontology, as indicated by the 80.7% of our respondents who agreed it would bring huge improvement to the specialty.

Such a positive perception has also been recorded from dentists in Turkey, at 85.70%^33^, and South Korea, at 64.2%^34^, and other populations^23,31,35^, reflecting a worldwide consensus on the potential role of AI, despite a variable acquaintance with the matter.

Some of the respondents (78.2%) disagreed that AI can replace periodontists in diagnosing periodontal diseases, but they feel that AI can successfully help in taking their dental practice to a new level, which is in agreement with previous studies^36–38^. Comparable findings were reported by Jeong et al.,^34^ and Yüzbaşıoğlu^33^, , where most participants did not agree that AI could replace them soon.

Regarding clinical applications, 58.5% of participants agreed with AI’s definitive role in periodontal disease prognosis prediction. Additionally, participants identified the use of AI for radiographic detection, diagnosis, and treatment planning, an observation that was consistent with findings in previous studies conducted in Turkey, Saudi Arabia, Egypt, Morocco, and India^18,23,26,31–33,39,40^.

Interest in receiving more training in AI applied to dentistry among periodontists was expressed by 53.1%. One possible explanation for this could be that the participants are eager to learn about new dental technology that has the potential to improve treatment outcomes. It is consistent with previous studies indicating an increasing interest shown by dental practitioners to receive AI-related training and integrate AI subjects into dental education^32–34,40–42^.

Concerning the reliability of the periodontal diagnosis provided by AI in this study, 65.5% of the participants agreed that it is not reliable to trust the diagnosis provided by AI alone. This concern may be associated with the current limitations in AI for reproducing complex clinical judgment, incorporating patient-specific variables, and replacing human interaction in clinical decision-making^26,33,43–45^. More work needs to be done to create awareness about both the abilities and limitations of AI among medical professionals, as found in our analysis^46^.

The recent objective assessments of the AI-generated dental information also support the significance of focusing on reliability and quality prior to applying AI solutions for practical purposes. For instance, Ceylan Şen et al.^47^ evaluated the differences between ChatGPT-4, DeepSeek-R1, Microsoft Copilot, and Google Gemini, which are four commonly used AI chatbots, concerning patient information on the failure of dental implants.

Despite having shown some promising results when it comes to being supportive tools in educating patients, the authors noted the necessity of specific instructions for particular models and their improvement before their use in routine clinical practice. In the same way, Gaş et al.^48^ studied the answers generated by the AI concerning external sinus lifting and concluded that different AI models vary in terms of reliability, quality, usefulness, and readability in clinical settings. All of the above-mentioned facts confirm the worries expressed by the participants in this study.

### Concerns and barriers to AI adoption

Concerns about data protection and confidentiality have already been identified, as 59.3% of the participants were concerned about the potential security risk of using AI in periodontology. Periodontists perceived an AI application as not fully safeguarded against data protection breaches or legal conflicts, which was a clear barrier. Another barrier was related to the previous point. Both patients and dentists feared errors of the AI not being identifiable and leading to wrong therapeutic decisions^49^.

Ethical issues, including the risk of bias and transparency issues, appeared as recurring major concerns^50^. In our study, 52.7% of the participants were concerned that the use of AI would be a violation of patient privacy. There could be a couple of reasons for this, like periodontists are not yet completely familiar with the concept and are still unfamiliar with the true potential areas. There are still many obstacles to overcome since people are still reluctant to trust the outcomes that AI can provide in robot-based periodontal surgery^51,52^.

#### Factors associated with AI knowledge, perception, and usage

Multivariate ordinal logistic regression analyses were performed to explore the demographic and professional factors that were independently associated with different dimensions of AI engagement among Egyptian periodontists.

#### Factors associated with AI knowledge

Awareness of AI working principles was significantly associated with age category and professional experience (OR = 32.33, *P* = 0.002 and OR = 11.61, *P* = 0.009, respectively).

This finding suggests that AI awareness might be related to differences in digital exposure, professional development opportunities, and accumulated clinical experience among periodontists^53–55^. Gender, level of education, and institutional affiliation were not significantly associated factors.

#### Factors associated with AI perception

Gender was significantly associated with the perception that AI might replace periodontists, in which males had almost eight times the odds of this perception (OR = 7.87, *p* = 0.026) compared to females. The gender variations in risk perception in relation to technological change have been observed in other research concerning the adoption of new technology in the medical field^56,57^. This reflects the overall dental professionals’ perception worldwide in recognizing the benefits of AI but doubting its autonomy^36,37^.

#### Factors associated with AI use

Attendance in AI-related educational activities was significantly associated with age category, professional experience, and institutional affiliation. However, due to the coding direction of the regression model, the direction of these associations should be interpreted cautiously.

Such results imply the possibility of the existence of potential differences in AI educational engagement based on demographic and professional factors, which aligns with the previous evidence showing that factors such as career stage, digital experience, and organizational context can affect healthcare professionals’ adoption of AI technology^31,58^.

#### Factors associated with AI concerns

Gender was significantly associated with AI-related concerns, with men reporting lower levels of concern regarding AI diagnostic accuracy. This finding aligns with the gender-related differences observed in AI perception. Regarding privacy, the level of education (PhD) was associated with higher levels of concern, indicating that higher levels of educational sophistication may enhance understanding of associated ethics and implications of data protection/integrity^59,60^. This confirms the prevailing view that AI concerns in healthcare are differentiated based on inherent demographics and intellectual expertise^61^.

#### Synthesis and implications

Taken together, the regression analyses indicate that the engagement with AI among Egyptian periodontists is a complex function of the interactions among the variables of age, experience, male vs. female, and institutional setting. The associations observed between age, professional experience, and AI knowledge can provide guidelines on how to incorporate AI into undergraduate and postgraduate courses and how to create continuing education programs for experienced periodontists. The male-female differential perception and issues pointed out through the regression analysis warrant the imperative of differentiated strategies for the implementation of AI programs. The institutional differences observed in AI-related engagement may provide an opportunity for interinstitutional networks.

#### Strengths of the study

This research has a number of important advantages. To the best of the authors’ knowledge, it is the first national multicenter study conducted in Egypt and the ME region as a whole addressing AI knowledge, perception, use, and concerns directed at periodontists who practice a highly innovative branch of medicine. It had a large sample size (*n* = 275) with adequate statistical power, supporting the reliability of the performed analyses. Additionally, the use of a well-tested multidimensional instrument enabled a complex evaluation that went beyond mere knowledge. Finally, the employment of ordinal logistic regression analysis enabled a subtle exploration of the distinct roles of the demographic and professional factors with a clear-cut estimation of key parameters of AI adoption instead of being restricted to mere reporting.

#### Limitations

The current study has multiple limitations. First, its cross-sectional design and reliance on self-reported data preclude causal inferences, and response bias may be present. Secondly, the selection and self-selection biases associated with convenience sampling and professional network recruiting could have influenced the results, as those interested in AI were more likely to participate, which may limit the generalizability of the results. Thirdly, the study assessed perceptions and awareness rather than clinical outcomes or the effectiveness of AI tools in real-world practice. Fourth, the study evaluated self-reported AI usage rather than objectively verified use of particular AI applications, which may overestimate or underestimate actual engagement. Moreover, in some cases, the regression estimates exhibited wide confidence intervals, which may reflect uncertainty in the precision of such associations; therefore, these findings should be interpreted cautiously, and further research with increased sample sizes is needed. Finally, while the official language of instruction at the participating dental faculties is English, conducting the survey exclusively in English could lead to suboptimal understanding for some individuals who are not native speakers.

#### Educational implications

As can be seen from the results of this study, there is a significant disparity between knowledge and application of AI among medical practitioners, thus pointing out the inadequacy of educational systems to convert theoretical knowledge into practice. Therefore, AI training must be integrated within postgraduate courses in dentistry, paying attention to hands-on activities and clinically meaningful cases related to periodontology.

Increasing digital literacy and skills associated with AI technology use will help to ensure more competent use of these tools in the diagnosis and treatment of patients. At the same time, the presence of associations involving factors like age and professional experience among associated factors indicates the importance of targeting specific groups of learners.

In addition to this, CPD programs need to adapt to changes due to the fast-paced development of artificial intelligence technology. Through appropriate CPD programs, it may be possible to compensate for a lack of confidence and competence with regard to the use of AI by practitioners. Critical evaluation skills and evidence-based application of artificial intelligence will play a key role in this.

#### Future prospects

Future research needs to move from perception assessment to evaluation of real-world clinical impact, cost-effectiveness, and long-term reliability using longitudinal, multicenter, and randomized controlled studies. There is a simultaneous, pressing need for collaboration across clinicians, universities, technology developers, and regulatory bodies. Such collaborations should focus on the development of robust frameworks for standardized data governance, transparent AI validation, and clear ethical guidelines. These efforts are crucial for building trust, ensuring patient safety, and facilitating the equitable and responsible integration of AI into periodontal practice.

## Conclusions

The current study offers a national overview of AI adoption among periodontists in Egypt, demonstrating a clear gap between high awareness and limited clinical implementation of AI technologies despite a favorable attitude towards them. Age, professional experience, gender, and institutional affiliation were identified as factors associated with AI-related knowledge, perception, and usage within the studied population.

In view of such findings, there is a need to integrate structured AI education within both postgraduate curricula and continuing professional development programs to address the identified educational deficiencies and promote evidence-based integration of AI technologies. Closing such a gap may contribute to improving preparedness for the appropriate adoption and proper use of AI technology within the field of periodontics.

## Supplementary Information

Below is the link to the electronic supplementary material.

Supplementary Material 1

Supplementary Material 2

Supplementary Material 3

Supplementary Material 4

Supplementary Material 5

## Data Availability

Research data supporting this publication is available from the corresponding author upon request.

## Abbreviations

AI: Artificial Intelligence
ML: Machine Learning
NNs: Neural Networks
CPD: Continuing Professional Development
GBD: Global Burden of Disease
SD: Standard Deviation
SPSS: Statistical Package for the Social Sciences
ANOVA: Analysis of Variance
IRB: Institutional Review Board (if referred to in methods/ethics section)
YLDs: Years Lived with Disability
AUC: Area Under the Curve

## References

[1] Abou-Bakr, A., El Barbary, A. & Hassanein, F. E. A. ChatGPT-5 vs oral medicine experts for rank-based differential diagnosis of oral lesions: a prospective, biopsy-validated comparison. *Odontology* (2025). 10.1007/s10266-025-01242-x PMC13319845 41247661

[2] Abou-Bakr, A. et al. *Comparative diagnostic accuracy of ChatGPT models in salivary gland disease: a multimodal vignette-based evaluation* (European Archives of Oto-Rhino-Laryngology, 2025). 10.1007/s00405-025-09925-5 41466068

[3] Hassanein, F. E. A. et al. Multimodal large language models for oral lesion diagnosis: a systematic review of diagnostic performance and clinical utility. *Frontiers Oral Health* Volume 7–2026. (2026). 10.3389/froh.2026.1748450 PMC12971682 41816099

[4] Roganović, J., Radenković, M. & Miličić, B. Responsible Use of Artificial Intelligence in Dentistry: Survey on Dentists’ and Final-Year Undergraduates’ Perspectives. In: *Healthcare* **11**; (2023). 10.3390/healthcare11101480 PMC10218709 37239766

[5] Robaian, A., Hassanein, F. E. A., Hassan, M. T., Alqahtani, A. S. & Abou-Bakr, A. A Multimodal Large Language Model Framework for Clinical Subtyping and Malignant Transformation Risk Prediction in Oral Lichen Planus: A Paired Comparison With Expert Clinicians. *Int. Dent. J.* **76** (1), 109357 (2026). 41477929 10.1016/j.identj.2025.109357 PMC12805022

[6] Hassanein, F. E. A. et al. Artificial Intelligence Versus Human Dental Expertise in Diagnosing Periapical Pathosis on Periapical Radiographs: A Multicenter Study. *Bioeng. vol*. **13**, 232 (2026). 10.3390/bioengineering13020232 PMC12937961 41749771

[7] Kim, E. H. et al. Prediction of Chronic Periodontitis Severity Using Machine Learning Models Based On Salivary Bacterial Copy Number. *Front. Cell. Infect. Microbiol.* **10**, 571515 (2020). 33304856 10.3389/fcimb.2020.571515 PMC7701273

[8] Ding, H. et al. Artificial intelligence in dentistry-A review. *Front. Dent. Med.* **4**, 1085251 (2023). 39935549 10.3389/fdmed.2023.1085251 PMC11811754

[9] Scott, J., Biancardi, A. M., Jones, O. & Andrew, D. Artificial Intelligence in Periodontology: A Scoping Review. *Dent J. (Basel)* **11**(2). (2023). 10.3390/dj11020043 PMC9955396 36826188

[10] Machoy, M. E., Szyszka-Sommerfeld, L., Vegh, A., Gedrange, T. & Woźniak, K. The ways of using machine learning in dentistry. *Adv. Clin. Exp. Med.* **29** (3), 375–384 (2020). 32207586 10.17219/acem/115083

[11] Jundaeng, J., Chamchong, R. & Nithikathkul, C. Artificial intelligence-powered innovations in periodontal diagnosis: a new era in dental healthcare. *Front. Med. Technol.* **6**, 1469852 (2024). 39866670 10.3389/fmedt.2024.1469852 PMC11757292

[12] Hassanein, F. E., Ibrahim, S. S., Tomo, S., Alsahhaf, A. & Abou-Bakr, A. Prompt engineering shapes diagnostic accuracy and explanation quality of LLM in oral lesion diagnosis: a prospective, expert-blinded benchmark study. *Odontology* **2026**:1–14 . 10.1007/s10266-026-01380-w 41966636

[13] Hassanein, F. E., Hassan, M. T., Jahngir, M., Javed, M. & Abou-Bakr, A. Abstraction-dependent diagnostic performance of a multimodal foundation model in oral epithelial dysplasia. *Odontology* **2026**:1–14 . 10.1007/s10266-026-01402-7 42115485

[14] Tai, M. C. The impact of artificial intelligence on human society and bioethics. *Tzu Chi Med. J.* **32** (4), 339–343 (2020). 33163378 10.4103/tcmj.tcmj_71_20 PMC7605294

[15] Hassanein, F. E. et al. Calibration of AI large language models with human subject matter experts for grading of clinical short-answer responses in dental education. *BMC Oral Health* (2026). 10.1186/s12903-026-07665-4 PMC12896245 41652418

[16] Hassanein, F. E., El-Guindy, J., Ahmed, Y. & Abou-Bakr, A. Evaluating Multimodal Large Language Models for Clinical Diagnosis of Oral Lesions: A Biomedical Informatics Perspective. In: *Twelfth International Conference on Intelligent Computing and Information Systems (ICICIS): 2025*: IEEE; 2025: 552–559.: IEEE; 2025: 552–559. (2025).

[17] Chatzopoulos, G. S., Koidou, V. P., Tsalikis, L. & Kaklamanos, E. G. Clinical Applications of Artificial Intelligence in Periodontology: A Scoping Review. In: *Medicina* **61**; (2025). 10.3390/medicina61061066 PMC12195203 40572754

[18] Dashti, M. et al. Attitudes, knowledge, and perceptions of dentists and dental students toward artificial intelligence: a systematic review. *J. Taibah Univ. Med. Sci.* **19** (2), 327–337 (2024). 38293587 10.1016/j.jtumed.2023.12.010 PMC10825554

[19] Aldakhil, S. et al. Awareness and Approaches Regarding Artificial Intelligence in Dentistry: A Scoping Review. *Cureus* **16** (1), e51825 (2024). 38327934 10.7759/cureus.51825 PMC10847710

[20] Yılmaz, C., Erdem, R. Z. & Uygun, L. A. Artificial intelligence knowledge, attitudes and application perspectives of undergraduate and specialty students of faculty of dentistry in Turkey: an online survey research. *BMC Med. Educ.* **24** (1), 1149 (2024). 39407168 10.1186/s12909-024-06106-6 PMC11481589

[21] Chawla, R. L. et al. Knowledge, Attitude and Perception Regarding Artificial Intelligence in Periodontology: A Questionnaire Study. *Cureus* **15** (11), e48309 (2023). 38058340 10.7759/cureus.48309 PMC10697475

[22] Robaian, A., Hamed, M. M., Ahmed, Y. & Hassanein, F. E. A. Comparative Evaluation of Customized CAD/CAM vs. Stock Titanium Abutments for Immediate Implant Placement in Class II Extraction Sockets: A Randomized Controlled Trial. *Dentistry J. vol*. **13**, 371 (2025). 10.3390/dj13080371 PMC12385633 40863074

[23] Elchaghaby, M. & Wahby, R. Knowledge, attitudes, and perceptions of a group of Egyptian dental students toward artificial intelligence: a cross-sectional study. *BMC Oral Health*. **25** (1), 11 (2025). 39754101 10.1186/s12903-024-05282-7 PMC11697735

[24] Sarker, I. H. AI-Based Modeling: Techniques, Applications and Research Issues Towards Automation, Intelligent and Smart Systems. *SN Comput. Sci.* **3** (2), 158 (2022). 35194580 10.1007/s42979-022-01043-x PMC8830986

[25] Sallam, M., Salim, N. A., Barakat, M. & Al-Tammemi, A. B. ChatGPT applications in medical, dental, pharmacy, and public health education: A descriptive study highlighting the advantages and limitations. *Narra J.* **3** (1), e103 (2023). 38450035 10.52225/narra.v3i1.103 PMC10914078

[26] Kansal, R. et al. Differences in Knowledge and Perspectives on the Usage of Artificial Intelligence Among Doctors and Medical Students of a Developing Country: A Cross-Sectional Study. *Cureus* **14** (1), e21434 (2022). 35223222 10.7759/cureus.21434 PMC8860704

[27] Price, W. N. 2, Gerke, S., Cohen, I. G. & nd, Potential Liability for Physicians Using Artificial Intelligence. *Jama* **322** (18), 1765–1766 (2019). 10.1001/jama.2019.15064 31584609

[28] Stogiannos, N., Georgiadou, E., Rarri, N. & Malamateniou, C. Ethical AI: A qualitative study exploring ethical challenges and solutions on the use of AI in medical imaging. *Eur. J. Radiol. Artif. Intell.* **1**, 100006 (2025).

[29] Swed, S. et al. Knowledge, attitude, and practice of artificial intelligence among doctors and medical students in Syria: A cross-sectional online survey. *Front. Artif. Intell.* **5**, 1011524 (2022). 36248622 10.3389/frai.2022.1011524 PMC9558737

[30] Althubaiti, A. Information bias in health research: definition, pitfalls, and adjustment methods. *J. Multidiscip Healthc.* **9**, 211–217 (2016). 27217764 10.2147/JMDH.S104807 PMC4862344

[31] Khanagar, S. et al. Knowledge, attitudes, and perceptions of dental students towards artificial intelligence in Riyadh. *Saudi Arabia*. **25**, 1857–1867 (2021).

[32] Murali, S. et al. Knowledge, attitude, and perception of dentists regarding the role of Artificial Intelligence and its applications in Oral Medicine and Radiology: a cross sectional study. *J. Oral Med. Oral Surg.* **29** (2), 22 (2023).

[33] Yüzbaşıoğlu, E. Attitudes and perceptions of dental students towards artificial intelligence. *J. Dent. Educ.* **85** (1), 60–68 (2021). 32851649 10.1002/jdd.12385

[34] Jeong, H., Han, S-S., Jung, H-I., Lee, W. & Jeon, K. J. Perceptions and attitudes of dental students and dentists in South Korea toward artificial intelligence: a subgroup analysis based on professional seniority. *BMC Med. Educ.* **24** (1), 430 (2024). 38649951 10.1186/s12909-024-05441-y PMC11034023

[35] Eroğlu Çakmakoğlu, E. & Günay, A. Dental Students’ Opinions on Use of Artificial Intelligence: A Survey Study. *Med. Sci. Monit.* **31**, e947658 (2025). 40302192 10.12659/MSM.947658 PMC12051404

[36] Agrawal, P. & Nikhade, P. Artificial Intelligence in Dentistry: Past, Present, and Future. *Cureus* **14** (7), e27405 (2022). 36046326 10.7759/cureus.27405 PMC9418762

[37] Tandon, D. & Rajawat, J. Present and future of artificial intelligence in dentistry. *J. Oral Biol. Craniofac. Res.* **10** (4), 391–396 (2020). 32775180 10.1016/j.jobcr.2020.07.015 PMC7394756

[38] Sadighian, J., Wilson, K., Crawford, M. & Wong, C. Evolving Stark Effect During Growth of Perovskite Nanocrystals Measured Using Transient Absorption. *Front. Chem.* **8**, 585853 (2020). 33195083 10.3389/fchem.2020.585853 PMC7594514

[39] Aboalshamat, K. Perception and Utilization of Artificial Intelligence (AI) among Dental Professionals in Saudi Arabia. *The Open. Dentistry Journal* 16. (2022).

[40] Elhijazi, M. & Benyahya, I. Artificial intelligence in dentistry: Knowledge and perceptions of Moroccan students. *Health Sciences* 4. (2023).

[41] Kalaimani, G., B, S., Chockalingam, R. M. & Karthick, P. Evaluation of Knowledge, Attitude, and Practice (KAP) of Artificial Intelligence Among Dentists and Dental Students: A Cross-Sectional Online Survey. *Cureus* **15** (9), e44656 (2023). 37799215 10.7759/cureus.44656 PMC10549783

[42] Aldowah, O. et al. Perceptions and Knowledge of Undergraduate Dental Students about Artificial Intelligence in Dental Schools: A Cross-sectional Study. *J. Contemp. Dent. Pract.* **25** (2), 148–155 (2024). 38514412 10.5005/jp-journals-10024-3633

[43] Pringle, A. J., Kumaran, V., Missier, M. S. & Nadar, A. S. P. Perceptiveness and Attitude on the use of Artificial Intelligence (AI) in Dentistry among Dentists and Non-Dentists - A Regional Survey. *Journal Pharm. Bioallied Sciences* **16**(Suppl 2). (2024). 10.4103/jpbs.jpbs_1019_23 PMC11174187 38882768

[44] Akingbola, A., Adeleke, O., Idris, A., Adewole, O. & Adegbesan, A. Artificial Intelligence and the Dehumanization of Patient Care. *J. Med. Surg. Public. Health*. **3**, 100138 (2024).

[45] Chan, C. K. AI as the Therapist: Student Insights on the Challenges of Using Generative AI for School Mental Health Frameworks. In: *Behavioral Sciences.* vol. 15; (2025). 10.3390/bs15030287 PMC11939552 40150182

[46] Montemayor, C., Halpern, J. & Fairweather, A. In principle obstacles for empathic AI: why we can’t replace human empathy in healthcare. *AI Soc.* **37** (4), 1353–1359 (2022). 34054228 10.1007/s00146-021-01230-z PMC8149918

[47] ŞenSC et al. Quality and Reliability of AI Information on Dental Implant Failure: A Comparative Multi-Model Analysis. *J. Craniofac. Surg.* 10.1097 (2026). 10.1097/SCS.0000000000012415 41553071

[48] Gaş, S., Uçan, G. Ö., Akcan, S. K., Paksoy, T. & Uncu, S. A. From Algorithm to Operatory: How Reliable Is Artificial Intelligence in Educating Patients on External Sinus Lifting? *J. Oral Maxillofac. Surg.* **84** (5), 732–745 (2026). 41592745 10.1016/j.joms.2026.01.001

[49] Müller, A., Mertens, S. M., Göstemeyer, G., Krois, J. & Schwendicke, F. Barriers and Enablers for Artificial Intelligence in Dental Diagnostics: A Qualitative Study. *J Clin. Med* **10**(8). (2021). 10.3390/jcm10081612 PMC8069285 33920189

[50] Sallam, M. ChatGPT Utility in Healthcare Education, Research, and Practice: Systematic Review on the Promising Perspectives and Valid Concerns. *Healthcare (Basel)* **11**(6). (2023). 10.3390/healthcare11060887 PMC10048148 36981544

[51] Jena, A. et al. Knowledge and Perception of Artificial Intelligence Amidst the Health Professionals-A Web-Based Survey. *J. Pharm. Bioallied Sci.* **16** (Suppl 5), S4365–s4367 (2024). 40061758 10.4103/jpbs.jpbs_647_24 PMC11888686

[52] Singh, N., Pandey, A., Tikku, A. P., Verma, P. & Singh, B. P. Attitude, perception and barriers of dental professionals towards artificial intelligence. *J. Oral Biol. Craniofac. Res.* **13** (5), 584–588 (2023). 37576799 10.1016/j.jobcr.2023.06.006 PMC10415790

[53] Imran, M., Almusharraf, N. & Abbasova, M. Y. Digital learning transformation: A study of teachers’ post-Covid-19 experiences. *Social Sci. Humanit. Open.* **11**, 101228 (2025).

[54] Neter, E., Western, M. J., Cooper, R., Silva, A. M. & König, L. M. Towards bridging the digital divide: training healthcare professionals for digitally inclusive healthcare systems. *Global Health Res. Policy*. **10** (1), 31 (2025). 10.1186/s41256-025-00433-x PMC12309028 40739280

[55] Greenhalgh, T. et al. Beyond Adoption: A New Framework for Theorizing and Evaluating Nonadoption, Abandonment, and Challenges to the Scale-Up, Spread, and Sustainability of Health and Care Technologies. *J. Med. Internet Res.* **19** (11), e367 (2017). 29092808 10.2196/jmir.8775 PMC5688245

[56] Carter, L. Exploring the Intersection of the Digital Divide and Artificial Intelligence: A Hermeneutic Literature Review. *AIS Trans. Hum Comput Interaction* (2020).

[57] Scheetz, J. et al. A survey of clinicians on the use of artificial intelligence in ophthalmology, dermatology, radiology and radiation oncology. *Sci. Rep.* **11** (1), 5193 (2021). 33664367 10.1038/s41598-021-84698-5 PMC7933437

[58] Joda, T. et al. Recent Trends and Future Direction of Dental Research in the Digital Era. In: *International Journal of Environmental Research and Public Health.* vol. 17; : 1987. (2020). 10.3390/ijerph17061987 PMC7143449 32197311

[59] Murdoch, B. Privacy and artificial intelligence: challenges for protecting health information in a new era. *BMC Med. Ethics*. **22** (1), 122 (2021). 34525993 10.1186/s12910-021-00687-3 PMC8442400

[60] Gerke, S., Minssen, T. & Cohen, G. Chap. 12 - Ethical and legal challenges of artificial intelligence-driven healthcare. In: *Artificial Intelligence in Healthcare.* edn. Edited by Bohr A, Memarzadeh K: Academic Press; : 295–336. (2020).

[61] Asan, O., Bayrak, A. E. & Choudhury, A. Artificial Intelligence and Human Trust in Healthcare: Focus on Clinicians. *J. Med. Internet Res.* **22** (6), e15154 (2020). 32558657 10.2196/15154 PMC7334754

