# Supplementary material for: Educational gaps and factors associated with artificial intelligence adoption among Egyptian periodontists: a multicenter cross-sectional study
Source: Sci Rep. 2026 Aug 10;16:24715. doi: 10.1038/s41598-026-65434-3 (PMC13457601; doi:10.1038/s41598-026-65434-3)
Supplement: Supplementary file 1 [file 41598_2026_65434_MOESM1_ESM.docx]

**Supplementary File 1. Completed Checklist for Reporting Results of Internet E-Surveys (CHERRIES) for the online questionnaire survey**

| **CHERRIES checklist item** | **Explanation** | **Page number in manuscript** |
| --- | --- | --- |
| Describe survey design | Target population, sample frame, and type of sampling described (multicenter cross-sectional study; convenience sampling of Egyptian periodontists). | Page 4–5 |
| IRB approval | Ethical approval from Faculty of Dentistry, Ain Shams University (FDASU-RECIM012404). | Page 5 |
| Informed consent | Online informed consent obtained before questionnaire completion; anonymity and confidentiality maintained. | Page 5–6 |
| Data protection | Anonymous responses, no personal identifiers collected, restricted access to data by research team. | Page 6–7 |
| Development and testing | Questionnaire adapted from previous studies; pilot tested among 20 periodontists for clarity, feasibility, and comprehension. | Page 6 |
| Open survey versus closed survey | Survey distributed only to eligible Egyptian periodontists through invitations/professional networks. | Page 5–6 |
| Contact mode | Participants contacted electronically through emails and digital communication methods. | Page 5 |
| Advertising the survey | Survey distributed through Egyptian Society of Periodontology mailing list and postgraduate programs. | Page 6 |
| Web/E-mail | Electronic survey created using Google Forms and distributed online. | Page 6 |
| Context | Distributed through professional periodontology networks and academic institutions. | Page 5–6 |
| Mandatory/voluntary | Participation was voluntary and anonymous. | Page 6 |
| Incentives | No incentives or financial rewards were provided to participants for completing the survey. Participation was entirely voluntary. | Page 6 |
| Time/Date | Data collection period mentioned as academic year 2023–2024. | Page 4 |
| Randomization of items/questionnaires | No randomization performed. | N/A |
| Adaptive questioning | No adaptive questioning was applied. | N/A |
| Number of items | Questionnaire consisted of 33 questions. | Page 4, Page 7 |
| Number of screens/pages | Questionnaire sections available in Supplementary file. | Supplementary file |
| Completeness check | Mandatory responses were activated in Google Forms to avoid missing data. | Page 7–8 |
| Review step | Before final submission of the questionnaire, participants were able to review their responses and modify their answers if needed. | Page 7 |
| Unique site visitor | Not assessed. | N/A |
| View rate | Not calculated. | N/A |
| Participation rate | Not calculated. | N/A |
| Completion rate | Complete responses analyzed (275 responses included). | Page 5 |
| Cookies used | Cookies were not used to identify participants or track survey responses | Page 7 |
| IP check | IP addresses were not collected, stored, or used to identify participants | Page 7 |
| Log file analysis | Not performed. | N/A |
| Registration | No participant registration required. | Page 6–7 |
| Handling of incomplete questionnaires | Mandatory responses prevented incomplete questionnaires. | Page 7–8 |
| Questionnaires submitted with atypical timestamp | No time-based exclusion applied. | N/A |
| Statistical correction | No weighting or statistical correction for sample representativeness applied. | N/A |
