# Supplementary material for: Educational gaps and factors associated with artificial intelligence adoption among Egyptian periodontists: a multicenter cross-sectional study
Source: Sci Rep. 2026 Aug 10;16:24715. doi: 10.1038/s41598-026-65434-3 (PMC13457601; doi:10.1038/s41598-026-65434-3)
Supplement: Supplementary file 2 [file 41598_2026_65434_MOESM2_ESM.docx]

**Supplementary Table 1: Regression analysis of demographic and professional factors associated with understanding of AI working principles among Egyptian periodontists**

|  | **Multivariate** | | | | |
| --- | --- | --- | --- | --- | --- |
|  | **B** | **SE** | **Wald** | **p** | **OR (LL – UL 95% CI)** |
| **Sex [Male]** | 0.279 | 0.446 | 0.389 | 0.533 | 1.322 (0.550 – 3.171) |
| **Age (years)** |  |  |  |  | 1.000 |
| <30 | 3.476 | 1.106 | 9.874 | 0.002^*^ | 32.33 (3.699 – 282.591) |
| ≥30^®^ |  |  |  |  | 1.000 |
| **Level of education** |  |  |  |  |  |
| MSc | -0.951 | 0.554 | 2.942 | 0.086 | 0.386 (0.130 – 1.146) |
| PhD^®^ |  |  |  |  | 1.000 |
| **Number of years working in the profession** |  |  |  |  |  |
| ≤5^®^ |  |  |  |  | 1.000 |
| >5 | 2.452 | 0.936 | 6.856 | 0.009^*^ | 11.61 (1.853 – 72.748) |
| **Faculty** |  |  |  |  |  |
| Public universities^®^ |  |  |  |  | 1.000 |
| Private universities | 0.522 | 0.450 | 1.345 | 0.246 | 1.685 (0.698 – 4.076) |

B: Unstandardized Coefficient	OR: Odds ratio	SE: Estimates Standard error

CI: Confidence interval	LL: Lower limit	UL: Upper Limit

*: Statistically significant at p ≤ 0.05
