# Supplementary material for: Educational gaps and factors associated with artificial intelligence adoption among Egyptian periodontists: a multicenter cross-sectional study
Source: Sci Rep. 2026 Aug 10;16:24715. doi: 10.1038/s41598-026-65434-3 (PMC13457601; doi:10.1038/s41598-026-65434-3)
Supplement: Supplementary file 3 [file 41598_2026_65434_MOESM3_ESM.docx]

**Supplementary Table 2: Regression analysis of demographic and professional factors associated with perceptions of AI impact and future role in periodontology**

|  | **Perception that AI is a temporary trend** | | | | | **Perception that AI can improve the field of periodontology** | | | | | **Perception that AI could replace periodontists in the future** | | | | |
| --- | --- | --- | --- | --- | --- | --- | --- | --- | --- | --- | --- | --- | --- | --- | --- |
|  | **B** | **SE** | **Wald** | **p** | **OR (LL – UL 95%CI)** | **B** | **SE** | **Wald** | **p** | **OR(LL – UL 95% CI)** | **B** | **SE** | **Wald** | **p** | **OR(LL – UL 95% CI)** |
| **Sex [Male]** | 0.134 | 0.453 | 0.088 | 0.767 | 1.144 (0.471 – 2.780) | 0.772 | 0.830 | 0.863 | 0.353 | 2.163 (0.425 – 11.011) | 2.063 | 0.926 | 4.967 | 0.026^*^ | 7.867 (1.282 – 48.264) |
| **Age (years)** |  |  |  |  |  |  |  |  |  |  |  |  |  |  |  |
| <30 | -0.063 | 0.593 | 0.011 | 0.916 | 0.939 (0.294 – 3.003) | -0.955 | 0.640 | 2.228 | 0.136 | 0.385 (0.110 – 1.349) | -7.192 | 4.937 | 2.122 | 0.145 | 0.001(0.000004–12.003) |
| ≥30^®^ |  |  |  |  | 1.000 |  |  |  |  | 1.000 |  |  |  |  | 1.000 |
| **Level of education** |  |  |  |  |  |  |  |  |  |  |  |  |  |  |  |
| MSc | 0.225 | 0.482 | 0.219 | 0.640 | 1.253 (0.487 – 3.220) | 1.353 | 1.006 | 1.807 | 0.179 | 3.869 (0.538 – 27.813) | 0.001 | 0.747 | 0.000 | 0.999 | 1.001 (0.232 – 4.325) |
| PhD^®^ |  |  |  |  | 1.000 |  |  |  |  | 1.000 |  |  |  |  | 1.000 |
| **Number of years working in the profession** |  |  |  |  |  |  |  |  |  |  |  |  |  |  |  |
| ≤5^®^ |  |  |  |  | 1.000 |  |  |  |  | 1.000 |  |  |  |  | 1.000 |
| >5 | 0.907 | 0.511 | 3.153 | 0.076 | 2.478 (0.910 – 6.746) | -0.243 | 0.604 | 0.163 | 0.687 | 0.784 (0.240 – 2.560) | -2.132 | 1.359 | 2.458 | 0.117 | 0.119 (0.008 – 1.704) |
| **Faculty** |  |  |  |  |  |  |  |  |  |  |  |  |  |  |  |
| Public universities^®^ |  |  |  |  | 1.000 |  |  |  |  | 1.000 |  |  |  |  | 1.000 |
| Private universities | -0.080 | 0.444 | 0.033 | 0.856 | 0.923 (0.387 – 2.202) | -1.088 | 0.698 | 2.432 | 0.119 | 0.337 (0.086 – 1.322) | 0.713 | 0.579 | 1.514 | 0.219 | 2.039 (0.655 – 6.347) |

B: Unstandardized Coefficient	OR: Odds ratio	SE: Estimated standard error

CI: Confidence interval	LL: Lower limit	UL: Upper Limit

*: Statistically significant at p ≤ 0.05
