# Supplementary material for: Educational gaps and factors associated with artificial intelligence adoption among Egyptian periodontists: a multicenter cross-sectional study
Source: Sci Rep. 2026 Aug 10;16:24715. doi: 10.1038/s41598-026-65434-3 (PMC13457601; doi:10.1038/s41598-026-65434-3)
Supplement: Supplementary file 4 [file 41598_2026_65434_MOESM4_ESM.docx]

**Supplementary Table 3: Regression analysis of demographic and professional factors associated with AI-related educational engagement and use of AI-based dental applications**

|  | **Participation in AI-related educational activities** | | | | | **Familiarity with AI-based dental software applications** | | | | | **Use of AI for research purposes in periodontics** | | | | |
| --- | --- | --- | --- | --- | --- | --- | --- | --- | --- | --- | --- | --- | --- | --- | --- |
|  | **B** | **SE** | **Wald** | **p** | **OR(LL – UL 95%CI)** | **B** | **SE** | **Wald** | **p** | **OR(LL – UL 95%CI)** | **B** | **SE** | **Wald** | **p** | **OR(LL – UL 95%CI)** |
| **Sex [Male]** | -0.250 | 0.370 | 0.456 | 0.500 | 0.779 (0.377 – 1.609) | 0.159 | 0.488 | 0.106 | 0.745 | 1.172 (0.450 – 3.052) | 0.296 | 0.353 | 0.704 | 0.401 | 1.344 (0.674 – 2.683) |
| **Age (years)** |  |  |  |  |  |  |  |  |  |  |  |  |  |  |  |
| <30 | -2.682 | 0.952 | 7.934 | 0.005^*^ | 0.068 (0.011 – 0.442) | -0.279 | 0.524 | 0.282 | 0.595 | 0.757 (0.271 – 2.114) | -1.943 | 0.720 | 7.289 | 0.007^*^ | 0.143 (0.035 – 0.587) |
| ≥30^®^ |  |  |  |  | 1.000 |  |  |  |  | 1.000 |  |  |  |  | 1.000 |
| **Level of education** |  |  |  |  |  |  |  |  |  |  |  |  |  |  |  |
| MSc | 0.119 | 0.482 | 0.061 | 0.806 | 1.126 (0.438 – 2.898) | 0.996 | 0.721 | 1.908 | 0.167 | 2.707 (0.659 – 11.124) | -0.363 | 0.520 | 0.487 | 0.485 | 0.696 (0.251 – 1.927) |
| PhD^®^ |  |  |  |  | 1.000 |  |  |  |  | 1.000 |  |  |  |  | 1.000 |
| **Number of years working in the profession** |  |  |  |  |  |  |  |  |  |  |  |  |  |  |  |
| ≤5^®^ |  |  |  |  | 1.000 |  |  |  |  | 1.000 |  |  |  |  | 1.000 |
| >5 | -1.634 | 0.664 | 6.054 | 0.014^*^ | 0.195 (0.053 – 0.717) | 0.853 | 0.669 | 1.626 | 0.202 | 2.346 (0.633 – 8.703) | -1.600 | 0.609 | 6.913 | 0.009^*^ | 0.202 (0.061 – 0.665) |
| **Faculty** |  |  |  |  |  |  |  |  |  |  |  |  |  |  |  |
| Public universities^®^ |  |  |  |  | 1.000 |  |  |  |  | 1.000 |  |  |  |  | 1.000 |
| Private universities | -0.926 | 0.425 | 4.757 | 0.029^*^ | 0.396 (0.172 – 0.910) | -0.520 | 0.489 | 1.131 | 0.288 | 0.594 (0.228 – 1.551) | 0.312 | 0.346 | 0.811 | 0.368 | 1.366 (0.693 – 2.693) |

B: Unstandardized Coefficient	OR: Odds ratio	SE: Estimates Standard error

CI: Confidence interval	LL: Lower limit	UL: Upper Limit

*: Statistically significant at p ≤ 0.05
