# Supplementary material for: Educational gaps and factors associated with artificial intelligence adoption among Egyptian periodontists: a multicenter cross-sectional study
Source: Sci Rep. 2026 Aug 10;16:24715. doi: 10.1038/s41598-026-65434-3 (PMC13457601; doi:10.1038/s41598-026-65434-3)
Supplement: Supplementary file 5 [file 41598_2026_65434_MOESM5_ESM.docx]

**Supplementary Table 4: Regression analysis of demographic and professional factors associated with AI diagnostic reliability, patient privacy, security, and professional dependency concerns**

|  | **Concern regarding reliability of AI-assisted periodontal diagnosis** | | | | | **Concern regarding patient privacy when using AI** | | | | |
| --- | --- | --- | --- | --- | --- | --- | --- | --- | --- | --- |
|  | **B** | **SE** | **Wald** | **p** | **OR (LL – UL 95%CI)** | **B** | **SE** | **Wald** | **p** | **OR (LL – UL 95%CI)** |
| **Sex [Male]** | -1.623 | 0.747 | 4.728 | 0.030^*^ | 0.197 (0.046 – 0.852) | -0.565 | 0.324 | 3.040 | 0.081 | 0.568 (0.301 – 1.073) |
| **Age (years)** |  |  |  |  |  |  |  |  |  |  |
| <30 | 0.565 | 0.761 | 0.553 | 0.457 | 1.760 (0.396 – 7.818) | -0.313 | 0.405 | 0.598 | 0.439 | 0.731 (0.331 – 1.616) |
| ≥30^®^ |  |  |  |  | 1.000 |  |  |  |  | 1.000 |
| **Level of education** |  |  |  |  |  |  |  |  |  |  |
| MSc | -1.141 | 0.794 | 2.066 | 0.151 | 0.320 (0.067 – 1.514) | -1.564 | 0.648 | 5.831 | 0.016 | 0.209 (0.059 – 0.745) |
| PhD^®^ |  |  |  |  |  |  |  |  |  | 1.000 |
| **Number of years working in the profession** |  |  |  |  |  |  |  |  |  |  |
| ≤5^®^ |  |  |  |  | 1.000 |  |  |  |  | 1.000 |
| >5 | 3.239 | 1.994 | 2.638 | 0.104 | 25.50 (0.512 – 1269.5) | 0.241 | 0.599 | 0.162 | 0.687 | 1.273 (0.393 – 4.121) |
| **Faculty** |  |  |  |  |  |  |  |  |  |  |
| Public universities^®^ |  |  |  |  | 1.000 |  |  |  |  | 1.000 |
| Private universities | 1.096 | 0.766 | 2.049 | 0.152 | 2.991 (0.667 – 13.412) | 0.584 | 0.349 | 2.791 | 0.095 | 1.793 (0.904 – 3.555) |

B: Unstandardized Coefficient	OR: Odds ratio	SE: Standard error of the estimate

CI: Confidence interval	LL: Lower limit	UL: Upper Limit

*: Statistically significant at p ≤ 0.05
